# Dataset on the life cycle assessment of the production of stabilized lactic acid bacteria

**DOI:** 10.1016/j.dib.2025.111541

**Published:** 2025-04-08

**Authors:** Maite Gagneten, Camille Quentier, Stéphanie Passot, Stéphanie Cenard, Fernanda Fonseca, Caroline Pénicaud

**Affiliations:** a Departamento de Industrias, Facultad de Ciencias Exactas y Naturales, ITAPROQ (UBA-CONICET), Universidad de Buenos Aires, Ciudad Autónoma de Buenos Aires, Argentina; b Université Paris-Saclay, INRAE, AgroParisTech, UMR SayFood, 91120 Palaiseau, France

**Keywords:** Freezing, Freeze-drying, Spray-drying, Fermentation, Fructo-oligosaccharides, Acidifying activity, Biological activity

## Abstract

Lactic acid bacteria are widely used in the food and pharmaceutical industries to produce fermented foods and probiotics. However, very little is known about the environmental impacts of their production processes. This dataset provides appropriate data related to the environmental assessment by Life Cycle Assessment of thirty scenarios of production processes to produce lactic acid bacteria concentrates at the pilot scale. Life Cycle Inventory (LCI) foreground data were collected during experiments performed in 2021 in Biosearch Life, a Kerry Group company (Granada, Spain). They were manually measured, registered with sensors (tap water, steam, compressed air, and electricity consumption), or found in the technical and scientific literature. Storage experiments and biological activity measurements were performed during 2021 and 2022 in AgroParisTech (Thiverval-Grignon, France). Background data came from the database Ecoinvent 3.9.1, completed by Agribalyse 3.0. LCI of the fructo-oligosaccharide (FOS) protectants' production was obtained from another data paper. Life Cycle Impact Assessments (LCIA) were computed with SimaPro v9.5 software (Pré consultant) with the “EF 3.0 Method (adapted) V1.00 / EF 3.0 normalization and weighting set” to obtain the midpoint indicators. The dataset contains all the inventory data (mass and energy flows, equipment) and the biological activity data. The Life Cycle Inventory data could be reused by scientists for future LCAs. The environmental impacts computed by Life Cycle Assessment could be reused by scientists or the food industry for eco-design or environmental labeling.

Specifications Table

**Table:** 

Subject	Environmental Science
Specific subject area	Environmental assessment of the production of stabilized lactic acid bacteria
Type of data	Tables Figures Raw and analyzed data
Data collection	LCI foreground data were manually measured and registered with sensors (tap water, steam, compressed air, and electricity consumption) or found in the technical and scientific literature. Background data came from the database Ecoinvent 3.9.1, completed by Agribalyse 3.0. The LCI of producing the fructo-oligosaccharide (FOS) protectants was obtained from Gerbino et al., 2022 [1]. Life Cycle Assessments were computed with SimaPro v9.5 software (Pré consultant) with the "EF 3.0 Method (adapted) V1.00 / EF 3.0 normalization and weighting set" [2] to obtain the midpoint indicators.
Data source location	For bacteria production, protection, and stabilization: Institution: Biosearch Life, a Kerry Group Company City/Town/Region: Granada Country: Spain For biological activity and storage experiments: Institution: Université Paris-Saclay, INRAE, AgroParisTech, UMR SayFood City/Town/Region: Thiverval-Grignon Country: France Completion with literature data and LCA computing: Institution: Université Paris-Saclay, INRAE, AgroParisTech, UMR SayFood City/Town/Region: Palaiseau Country: France The background data from the Ecoinvent database were those for European consumption and production systems.
Data accessibility	Repository name: Data INRAE Data identification number: 10.57745/HTC3UB Direct URL to data: 10.57745/HTC3UB
Related research article	[3] M. Gagneten, C. Quentier, S. Passot, S. Cenard, F. Fonseca, C. Pénicaud, Joining environmental impacts and product quality in Life Cycle Assessment: The case of the production and storage of lactic acid bacteria concentrates, Cleaner Environmental Systems 15 (2024) 100245. 10.1016/j.cesys.2024.100245

## 1 Value of the Data

• This dataset presents LCIs (Life Cycle Inventories) and LCIAs (Life Cycle Impact Assessments) of thirty different scenarios of production and storage of stabilized lactic acid bacteria.
• This dataset presents the results of acidifying activity loss of two different lactic acid bacteria strains protected and stabilized by diverse strategies.
• It covers a wide range of production strategies, making it possible to compare those alternatives meaningfully.
• The Life Cycle Inventory data, Life Cycle Impact Assessment results, and Specific acidifying activity in this dataset provide transparency to the analysis described in the accompanying article [3].
• The Life Cycle Inventory data in this work could be reused by scientists for future LCAs, either in part for certain stages such as fermentation for biotechnological biomolecule productions or for the entire route with bacteria as ingredients of fermented foods (e.g., cheese, yogurt or their plant substitutes).
• The food industry could reuse the Life Cycle Impact Assessment results to design eco-friendly production strategies and environmental labeling.

## 2 Background

The dataset supports a research article, "Joining Environmental Impacts and Product Quality in Life Cycle Assessment: the Case of the Production and Storage of Lactic Acid Bacteria Concentrates.” Lactic acid bacteria are widely used in the food and pharmaceutical industries to produce fermented foods and probiotics. However, very little is known about the environmental impacts of these production processes. The dataset details life cycle inventories for thirty scenarios of production and storage of lactic acid bacteria concentrates at the pilot scale. The environmental impacts computed by Life Cycle Assessment are also provided to give quantitative environmental data for scientists and the food industry. These environmental impacts are provided for a mass-based functional unit. The biological activity of the bacteria is also provided to make it possible to have a joint environment and product quality analysis. The dataset thus aims to provide data useful for eco-design or environmental labeling.

## 3 Data Description

All inventory data (LCI) of stabilized lactic acid bacteria production and their environmental impacts (LCIA) results are available in the associated dataset. The dataset contains seven files:

1. Dataset_variables: Variable name and description, their unit (when relevant), the associated data type, and the allowed values for discrete data. All the variables used in the dataset are presented.
2. Dataset_stabilized_LAB_inventory: Inventory data for all production steps of the different stabilized lactic acid bacteria scenarios. The inventory includes all the flows taken into account, arranged by production steps. It indicates whether the flow is incoming or outgoing, its quantity, and the database and background data used to model it. This table is downstream to the “mediums&solutions” and the “equipment” tables. Data from this table must be aligned to the functional unit using the “alignment factors” table.
3. Dataset_stabilized_LAB_equipment: Data related to the equipment used, including the composition in nature and mass of each material and the power of the equipment to compute refrigerating liquid leakage in the case of refrigerated equipment. Each equipment is described for 1 p.
4. Dataset_stabilized_LAB_mediums&solutions: Inventory data for preparing the reagents, culture mediums, and cleaning solutions used during the different production steps described in Dataset_stabilized_LAB_inventory. The inventory includes all the flows taken into account, their quantity, and the database and background data used to model the flow. Each reagent, culture medium, and cleaning solution is described for 1 p produced.
5. Dataset_stabilized_LAB_alignment_factors: Factors to align the inventory data with the functional unit of 90 g of protected lactic acid bacteria (before stabilization).
6. Dataset_stabilized_LAB_LCIA: Life Cycle Impact Assessment of the production of 90 g of protected lactic acid bacteria (before stabilization) calculated using the characterization method “EF 3.0 Method (adapted) V1.00 / EF 3.0 normalization and weighting set”. The raw LCIA results for the functional unit without normalization or weighting are provided.
7. Dataset_t_spe_: Specific acidifying activity of the lactic acid bacteria after each production and storage step.

## 4 Experimental Design, Materials and Methods

For this study, we followed the steps of the standard LCA methodology [4].

### 4.1 Goal and scope

This LCA aimed to assess the environmental performance of stabilized lactic acid bacteria (LAB) production processes. The two main objectives were identifying the process steps contributing most to environmental degradation and comparing different production scenarios. LCA was performed using data collected at the laboratory scale to produce the fructo-oligosaccharides (FOS) [1] and data collected at the pilot scale to produce and stabilize the bacteria, supplemented with data from databases. The system considered all the processes involved in the production process, from the production of the inputs to the final stabilized LAB (“cradle to gate” approach), but transport, use, and final disposal were not included in the study. A functional unit of 90 g of protected LAB before stabilization was defined to allow comparison between the systems.

### 4.2 Description of the production scenarios of stabilized lactic-acid bacteria

Two LAB bacteria strains (*Lactobacillus delbrueckii* subsp. *bulgaricus* CFL1 and *Lactiplantibacillus plantarum* WCFS1), three fructo-oligosaccharides protective solutions (FOS 1, FOS 2 and FOS 6), and three different stabilization methods (freezing, freeze-drying and spray-drying) were addressed; and the cross-combination of them were studied. These resulted in thirty different production processes (scenarios) shown in Table 1. For each scenario, three alternatives in the duration of the storage were studied: 28 days, 90 days, and 365 days, leading to the study of 90 alternatives.

**Table 1:** Variations of the production processes and their combinations in the thirty production scenarios studied in this work.

LAB strain	FOS	Stabilization method	Storage temperature
*Lactobacillus delbrueckii* subsp. *bulgaricus* CFL1	FOS 1	Freezing	−80 °C
		Freeze-drying	4 °C 23 °C
		Spray-drying	4 °C 23 °C
	FOS 2	Freezing	−80 °C
		Freeze-drying	4 °C 23 °C
		Spray-drying	4 °C 23 °C
	FOS 6	Freezing	−80 °C
		Freeze-drying	4 °C 23 °C
		Spray-drying	4 °C 23 °C
*Lactiplantibacillus plantarum* WCFS1	FOS 1	Freezing	−80 °C
		Freeze-drying	4 °C 23 °C
		Spray-drying	4 °C 23 °C
	FOS 2	Freezing	−80 °C
		Freeze-drying	4 °C 23 °C
		Spray-drying	4 °C 23 °C
	FOS 6	Freezing	−80 °C
		Freeze-drying	4 °C 23 °C
		Spray-drying	4 °C 23 °C

Two independent replicates of each production process were conducted, and their corresponding environmental impact and biological quality were analyzed, except for *Lpb. plantarum* WCFS1 protected with FOS 2 and FOS 6. For *Lpb. plantarum* WCFS1 protected with FOS 2 and FOS 6, the Life Cycle Assessments were performed over one replicate due to practical limitations.

**Fig. 1:**
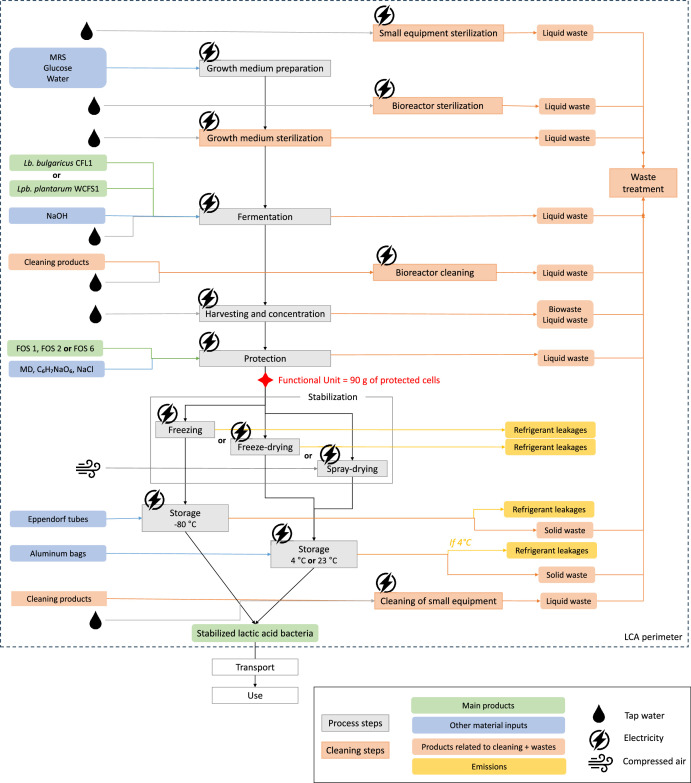
The production process of stabilized lactic acid bacteria. Production steps are in gray. The main products are green; the other input material products are blue, and the emissions are yellow. The tap water, electricity, and compressed air inputs are figured in pictograms. Cleaning and sterilization steps and their associated material inputs and outputs are in orange.

The production process consisted of six main steps described in Fig. 1 and detailed below: growth medium preparation, cell culture production, cell harvesting and concentration, protection, stabilization (freezing, freeze-drying, or spray-drying), and storage. All the experiments were performed on a pilot scale.

#### 4.2.1 Growth medium preparation and sterilization

The cell growth medium comprised 52 g/L MRS Broth and 20 g/L or 40 g/L of glucose for *Lb. bulgaricus* CFL1 and *Lpb. plantarum* WCFS1 respectively in deionized water (15 L). The whole medium was sterilized directly in the bioreactor at 120 °C for 20 min.

#### 4.2.2 Cell culture (fermentation)

Cultures were grown in a 30 L fermenter at 42 °C and 100 rpm agitation for *Lb. bulgaricus* CFL1 and 37 °C and 200 rpm agitation for *Lpb. plantarum* WCFS1. The pH was adjusted to 5.8 during growth by adding a 17 or 20 % v/v NaOH solution for each bacteria, respectively. Eight independent fermentation procedures were performed to produce *Lb. bulgaricus* CFL1 cell cultures (CFL1 - Fermentation 1 to 8), and three to produce *Lpb. plantarum* WCFS1 (WCFS1 – Fermentation 1 to 3). Since the different experiments differed regarding tap water, electricity and NaOH solution consumption, and liquid waste generation, each experiment is detailed separately in the LCI files in the dataset.

#### 4.2.3 Cell harvesting and concentration

Cells were harvested at the end of the exponential bacterial growth phase, one hour after reaching the maximum acidifying rate. For this, the culture medium was introduced into bottles of 10 L and immersed in an ice bath for 30 min, and then the cells were separated by centrifugation at 15,900 g and 4 °C for 10 min. The supernatant was discarded.

#### 4.2.4 Protection

The concentrated cells were mixed in an ice and water bath with a protective solution in 1:2 cells:protective medium weight ratio. Three different protectant agents were studied: a commercial fructo-oligosaccharide (Orafti® P95, Beneo Orafti; Tienen, Belgium) (FOS 1), a fructo-oligosaccharide produced by enzymatic synthesis without purification (FOS 2) and a purified fructo-oligosaccharide produced by enzymatic synthesis (FOS 6). All the protective solutions were prepared in ultrapure water and consisted of 120 g/L of the corresponding FOS, 120 g/L of commercial maltodextrin (Glucidex 6®, dextrose equivalent (DE) of 6, Roquette; Lestrem, France), 20 g/L of sodium ascorbate and 9 g/L NaCl. Since the different experiments differed in electricity consumption, amount of protective medium used, and liquid waste (biowaste) generated, each experiment is detailed separately in the LCI files in the dataset.

#### 4.2.5 Stabilization

Three stabilization processes were tested: freezing, freeze-drying, and spray-drying.

*Freezing*

1 mL of the protected bacterial concentrate was placed in Eppendorf tubes (10 tubes per formulation) and frozen at −80 °C.

*Freeze-drying*

The protected bacterial concentrates were frozen at −80 °C in sterile Petri dishes (30 g per Petri dish, 3 Petri dishes per formulation) and transferred to pre-cooled shelves at −50 °C in a pilot-scale freeze-dryer (VirTis Genesis 35 L SQ EL-85, SP Scientific; Warminster, PA, USA). After a holding step of 1h at −50 °C, the chamber pressure was decreased to 0.1 mbar, and the shelf temperature was increased from −50 °C to −20 °C at a heating rate of 0.25 °C/min to initiate the primary drying (sublimation phase). After 44 h, the shelf temperature was increased to 25 °C at 0.25 °C/min to initiate the secondary drying (desorption phase) that lasted 10 h. The dried samples were packed in aluminum bags.

*Spray-drying*

The protected bacterial concentrates were spray-dried in a mini spray-dryer (B-290 Büchi, Flawil, Switzerland) at a constant air inlet temperature of 105 °C and an outlet temperature of 65 °C. Atomization was performed by compressed air at a pressure of 6 bar and an air flux of 800 L/h with a nozzle diameter of 0.7 mm and a feed rate of 0.156 L/h.

#### 4.2.6 Storage

Samples were stored for up to 1 month at −80 °C for the frozen samples or 4 and 23 °C for the dehydrated samples. The three scenarios were used to study the influence of biological activity (highly dependent on storage conditions) on the environmental assessment.

#### 4.2.7 Cleaning and sterilization

Cleaning small equipment mainly used to prepare culture media was carried out in a washing machine, and sterilization was done in autoclaves. The bioreactor was washed with water (31 L) at 60 °C and 200 rpm for 2 h and sterilized at 120 °C for 20 min.

#### 4.2.8 Bacteria quality measurements

The bacteria's quality was measured in terms of their biological activity. The culturability and the acidifying activity were quantified before and after freezing, after freeze- or spray-drying, and during storage. Before the analysis, frozen samples were thawed, and the dried samples were rehydrated in 0.9 % saline water. The amount of saline water added was calculated to reach the same dry matter of the protected bacterial suspension before freeze-drying.

Culturability: after serial dilutions in sterilized saline solution, the appropriate dilutions were inoculated in MRS agar (Biokar Diagnostics, France) in triplicate. After incubation for 24 h at 37 °C for *Lpb. plantarum* WCFS1 and 48 h at 42 °C under anaerobic conditions (GENbox96124, BioMérieux, Marcy l'Etoile, France) for *Lb. bulgaricus* CFL1, colonies were enumerated from those plates containing between 30 and 300 colonies. The colony-forming units (CFU) were then multiplied by the dilution factor to determine the original concentration of cells, and results were expressed in log(CFU/mL).

Acidifying activity: the acidifying activity was determined using the Cinac system (AMS, Guidonia, Italia), according to the method described by [5] with some modifications. A volume of 100 µl of *Lb. bulgaricus* CFL1 or 10 µl of *Lpb. plantarum* WCFS1 cell suspension or rehydrated sample were incubated in skim milk at 42 °C or in yeast extract medium (10 g/L) added with glucose (10 g/L) at 37 °C for each bacteria strain, respectively. The time necessary to reach a pH drop of 1.5 points (t_dpH=1.5_, in minutes) was used to characterize the acidifying activity of the samples.

Specific acidifying activity: the specific acidifying activity (t_spe_ in min/(log(CFU/mL)) was defined as the ratio of t_dpH=1.5_ to the corresponding cell culturability [6,7]. Thus, t_spe_ gives a more meaningful result of the LAB's biological activity, considering its physiological state and viability. The lower the t_spe_, the better the physiological state.

The loss of specific acidifying activity was expressed as the increase of t_spe_ (Δt_spe_), calculated as the t_spe_ after stabilization or storage minus t_spe_ before those operations. Moreover, since a linear relationship was observed between the t_spe_ and the storage time, the rate of loss of specific acidifying activity (in min/(log(CFU/mL))/day)) was computed for each scenario and was used to estimate the specific acidifying activity for up to 12 months of storage.

The raw data for the culturability and the acidifying activity measurements are not directly linked to this data paper since only the resulting t_spe_ values were needed for the LCA studies. However, this raw data can be found in the published dataset “Mechanistic study of the differences in lactic acid bacteria resistance to freeze- or spray-drying and storage” (10.57745/AAARUZ).

The resulting t_spe_ are presented in the Dataset_tspe file.

### 4.3 Inventory data

The foreground LCI comprises measured and calculated data as described in 4.3.1, 4.3.2, and 4.3.3. The background LCI is described in Section 4.3.4.

#### 4.3.1 Equipment

The nature of the materials that composed the different equipment was taken from the equipment data sheets, and the mass amount was obtained from the same data sheets or manually measured in the case of small equipment and packaging. For the LCI, a temporal allocation was considered for large equipment (bioreactor, freezers, freeze-dryer, spray-dryer, centrifuge, agitator, autoclaves, heat-sealer, washing machine, proofer, water bath) according to Eq. (1), considering a lifetime of 30 years. A number-of-use allocation factor was used for small equipment according to Eq. (2). For volume allocation in the storage rooms, Eq. (3) was used.

(1) $$Temporal\;allocation\;factor=\frac{Duration\;of\;use\;\left(h\right)}{Lifetime\;of\;the\;equipment\;\left(h\right)}$$

(2) $$Number\;of\;use\;allocation\;factor=\frac{Times\;used}{Total\;number\;of\;uses}$$

(3) $$\text{Volume}\;\text{allocation}\;\text{factor}=\frac{\text{Volume}\;\text{occupied}\;\left(\%\right)\;}{\text{Occupancy}\;\text{rate}\;\left(\%\right)}$$

The equipment data are provided in the Equipment_data file. The equipment used to produce the FOS protectants was taken from another dataset [1]. In the equipment file, no allocation factor was considered for this equipment to avoid confusion with equipment submitted to measurements in this study. However, adequate allocations were considered for this equipment when linked to the main inventory Dataset_stabilized_LAB_inventory through the number of pieces of equipment taken into account.

#### 4.3.2 Mass flows

The mass flows of the reactants (MRS broth, glucose, NaOH, protectants, cleaning products), wastes, and the amounts of bacteria at each step were manually measured during the production process. Sensors provided by RS for water and steam (RS Components SAS, Beauvais, France) and Krohne for compressed air (Krohne SAS, Romans-sur-Isère, France) were installed in the fermenter and the spray-dryer to measure the mass flows of cooling water, steam, and compressed air. Loss of refrigerant of the freezer, freeze-drier, and cold room storage were calculated as in [8] following Eq. (4).

(4) Lossofrefrigerant=Equipmentpower*Refrigerantcharge*Leakagerate

The equipment power and refrigerant charge were obtained from data sheets. The annual leakage rate of refrigerant contained in the equipment was assumed to be 15 % [9,10]. The resulting inventory data related to mass flows are available in LCI files in the dataset.

#### 4.3.3 Energy flows

Electricity consumption was measured during the experiments with sensors installed on the equipment. Sensors used on the fermenter and freeze-dryer were provided by LEM (Distrame, France) and by MONNIT for the freezers and the spray-dryer (Seoul, South Korea). The inventory data related to electrical consumptions are available in LCI files in the dataset.

#### 4.3.4 Background data

The inventory data corresponding to the production, protection, and stabilization inputs (ingredients, electricity, refrigerant, tap water) and outputs (refrigerant leakage, wastewater treatment process) were taken from the Ecoinvent database v3.9.1 using the cut-off system model completed by Agribalyse 3.0 for ingredients of the growth medium.

The pilot plant process was located in Spain, so the electricity generation and imports/exports from this country were considered, while tap and wastewater data from Europe was used. A Life Cycle Inventory of the three protectants was available from a previous study published in a data paper by Gerbino et al. [1].

#### 4.3.5 Combination of file data to build an inventory

The leading inventory file, Dataset_stabilized_LAB_inventory, has to be combined with other files to obtain the specific inventory for a specific scenario.

First, this main inventory refers to the Dataset_stabilized_LAB_mediums&solutions file. This file provides the inventory for one piece of each medium and solution. The number of pieces to consider is given in the leading inventory file.

Second, the leading inventory and the medium & solutions files refer to the Dataset_stabilized_LAB_equipment file. In this file, the inventory for one piece of equipment is provided. The leading inventory and the medium & solutions files give the number of pieces to consider.

Third, it is essential to note that these three files provide data for a complete production line that produced more than the reference flow. Then, it is necessary to align the different steps of the process with the functional unit (90 g of protected cells), which is also the reference flow. The factor that aligns the production steps to the reference flow is calculated by using Eq. (5).

(5) $$Alignment\;factor=\frac{\text{mass}\;\text{of}\;\text{the}\;\text{reference}\;\text{flow}}{total\;mass\;of\;collected\;cells\;after\;fermentation\;+\;total\;mass\;of\;FOS\;protectant}$$

The alignment factors are provided in the Dataset_stabilized_LAB_alignment_factors file. All the steps of a given scenario are associated with the same alignment factor except the stabilization and the storage steps.

No alignment factor was needed for stabilization by freezing because the data were directly collected and linked to the reference flow. The cell stabilization by freeze-drying or spray-drying necessitated more cells than the reference flow to work in normal conditions. Hence, more cells were stabilized, and the data related to freeze-drying and spray-drying have to be aligned following Eq. (6).

(6) $$Alignment\;factor=\frac{\text{mass}\;\text{of}\;\text{the}\;\text{reference}\;\text{flow}}{total\;mass\;of\;stabilized\;cells\;\left(measured\;before\;stabilization\right)}$$

Finally, the storage data were directly collected according to the reference flow. Hence, no alignment factor was necessary. However, since three duration alternatives were assessed (28 days, 90 days, and 365 days), the data related to a given duration were obtained using the alignment factor described in the Eq. (7).

(7) $$Alignment\;factor=\frac{assessed\;duration\;\left(28,\;90\;or\;365\;days\right)}{\text{standard}\;\text{duration}\;\left(28\;\text{days}\right)}$$

### 4.4 Impact assessment

The Life Cycle Impact Assessment (LCIA) was performed using SimaPro v9.5 software (Pré consultant) with the "EF 3.0 Method (adapted) V1.00 / EF 3.0 normalization and weighting set" [2]. All the midpoint impact categories available in this method were calculated: Climate change, Ozone depletion, Ionising radiation, Photochemical ozone formation, Particulate matter, Human toxicity (non-cancer), Human toxicity (cancer), Acidification, Eutrophication (freshwater), Eutrophication, (marine), Eutrophication (terrestrial), Ecotoxicity (freshwater), Land use, Water use, Resource use (fossils), Resource use (mineral and metals). The raw LCIA results for the functional unit, but without normalization nor weighting, are provided in the Dataset_stabilized_LAB_LCIA file.

### 4.5 Weighting method

To compare the environmental impacts of the different LABs in the related research article, the environmental impact scores were weighted by the specific acidifying activity (t_spe_) measured for each LAB after each process step: protection, stabilization, and storage, using Eq. 8. For the production steps related to the obtention of the LAB biomass (medium preparation, cleaning, sterilization, and fermentation), the environmental impact of these steps was multiplied by the t_spe_ value determined after protection. Also, since previous experience by the group suggested that no activity loss happened at −80 °C, no t_spe_ loss was considered for storage at −80 °C.

(8) $$Weighted\;score=Characterized\;impact\;value\;\times \;t_{spe}$$

A high t_spe_ value indicates a low performance of the bacterial starter and, therefore, large quantities of starter to be used to reach the target acidifying activity. Consequently, at equivalent LCIA results (characterized impact values), a scenario producing low-performance LAB concentrates (high t_spe_ value) will have a higher weighted score than a scenario with successful starters.

These weighted scores are not provided in a file but can easily be calculated from Dataset_stabilized_LAB_LCIA and Dataset_t_spe_ files using Eq. (8).

## Limitations

Two biological replicates of the whole process were performed for all the production scenarios. However, for *Lpb. plantarum* WCFS1 protected with FOS 2 and with FOS 6; although both biological replicates were analyzed regarding their biological quality, only one replicate was analyzed regarding its environmental impact due to practical constraints. Also, the functional unit of 90 g of protected LAB before stabilization did not consider the mass loss in the spray-drying process (sticking to the spray-dryer walls).

In addition, the reuse of the data should consider that it was collected on a pilot scale, which cannot be considered representative of an industrial scale in terms of environmental impacts.

## Ethics Statement

This work did not involve human subjects or laboratory animals or any data collected from social media platforms and, therefore, did not encounter any ethical issues.

## CRediT Author Statement

**Maite Gagneten**: Conceptualization, Methodology, Validation, Formal analysis, Investigation, Data curation, Writing - original draft, Visualization. **Camille Quentier**: Conceptualization, Methodology, Validation, Formal analysis, Investigation, Resources. **Stéphanie Passot**: Conceptualization, Investigation, Resources, Writing - review & editing. **Stéphanie Cenard**: Investigation, Resources. **Fernanda Fonseca**: Conceptualization, Investigation, Resources, Writing - review & editing, Funding acquisition. **Caroline Pénicaud**: Conceptualization, Methodology, Formal analysis, Writing - review & editing, Supervision, Project administration.

## Data Availability

DataverseLife Cycle Assessment of the production of stabilized lactic acid bacteria at pilot scale (Original data).
